# Supplementary material for: Prediction of Parkinson’s Disease Using Machine Learning Methods
Source: Biomolecules. 2023 Dec 8;13(12):1761. doi: 10.3390/biom13121761 (PMC10741603; doi:10.3390/biom13121761)
Supplement: Supplementary file 1 [file biomolecules-13-01761-s001.zip › biomolecules-2680513-supplementary.pdf]

biomolecules

Article

Prediction of Parkinson's Disease Using Machine Learning Methods

Jiayu Zhang [1], Wenchao Zhou [1], Hongmei Yu [1], Tong Wang [1], Xiaqiong Wang [2], Long Liu [1,*] and Yalu Wen [3,*]

[1] Department of Health Statistics, School of Public Health, Shanxi Medical University, No 56 Xinjian South Road, Yingze District, Taiyuan 030001, China; zjiayu0217@gmail.com (J.Z.); zhouwenchao157@gmail.com (W.Z.); yu@sxmu.edu.cn (H.Y.); tongwang@sxmu.edu.cn (T.W.)

[2] Department of Epidemiology and Biostatistics, Southeast University, 87 Ding Jiaqiao Road, Nanjing 210009, China; xiaqiong.wang@auckland.ac.nz

[3] Department of Statistics, University of Auckland, 38 Princes Street, Auckland Central, Auckland 1010, New Zealand

* Correspondence: biostat-ll@sxmu.edu.cn (L.L.); y.wen@auckland.ac.nz (Y.W.)

Supplementary Figures

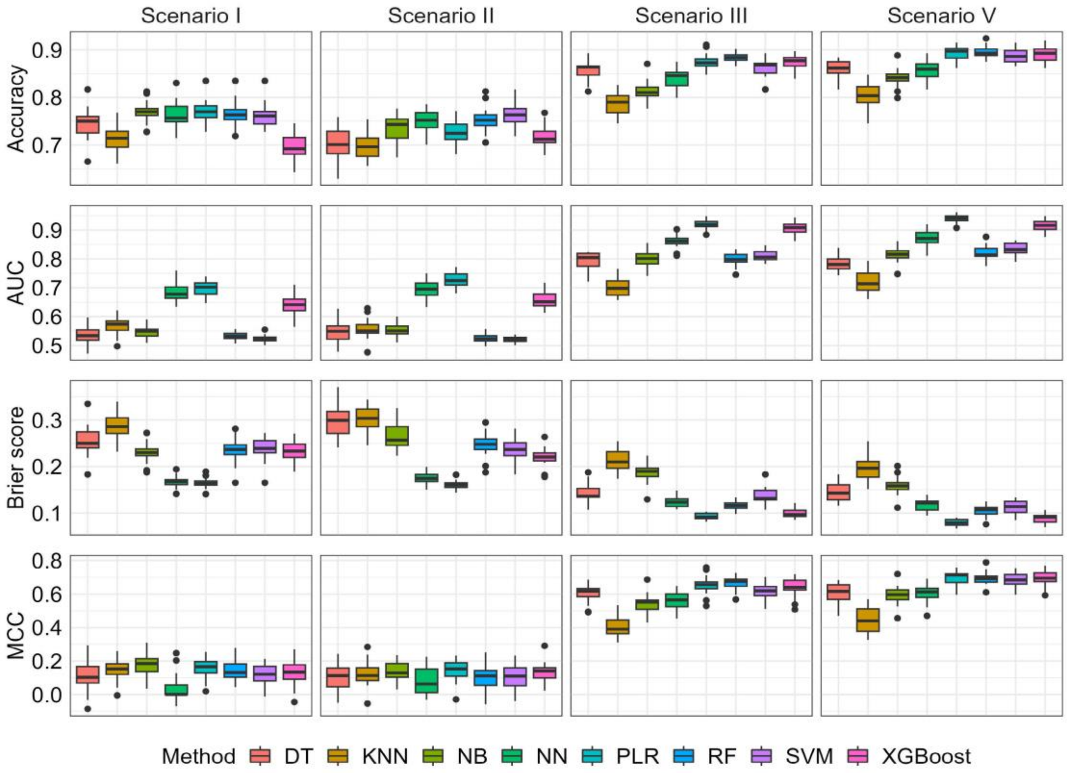

Figure S1. The comparisons of accuracy, area under the curve (AUC), Brier score, and Matthews Correlation Coefficient (MCC) among different ML methods. ML methods include decision tree (DT), K Nearest Neighbors (KNN), naïve Bayes (NB), neural network (NN), penalized logistic regression (PLR), random forest (RF), support vector machine (SVM), and Extreme Gradient Boosting (XGBoost). The scenarios assessed were as follows: Scenario I: demographic, Scenario II: demographic + hospital admission examinations, Scenario III: demographic + hospital admission examinations + clinical assessments, Scenario V: demographic + hospital admission examinations + clinical assessments + PRS + biomarkers.

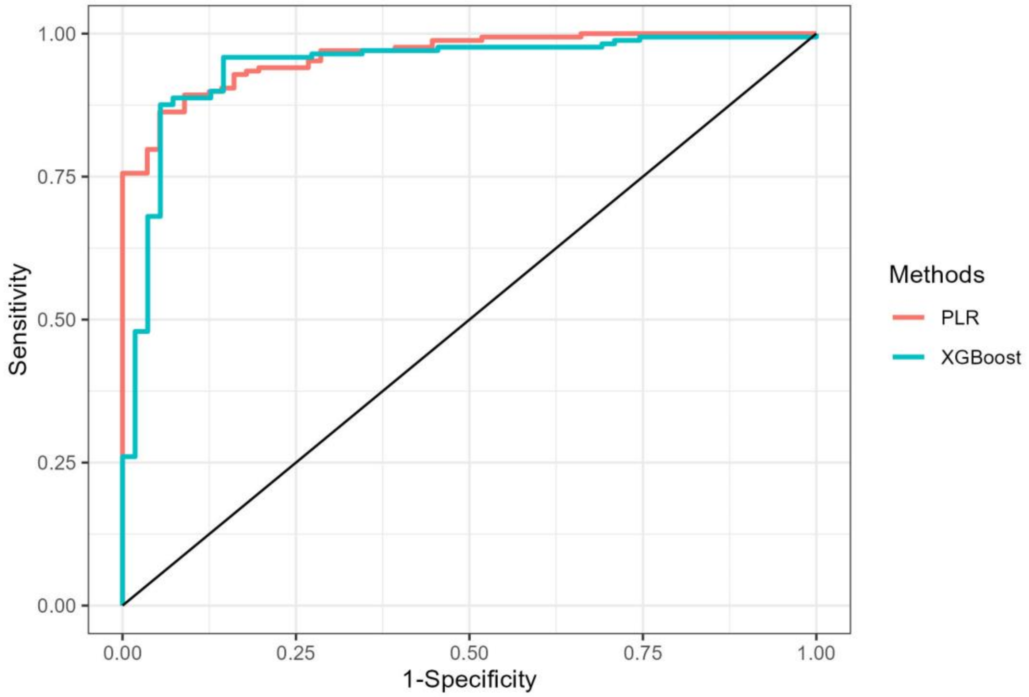

Figure S2. The Receiver Operating Characteristic curve that is derived from the best PD risk prediction models built by the penalized logistic regression and XGBoost methods. Predictors included in the models are demographic variables, hospital admission examinations, clinical assessments, and PRS.

Supplementary Tables

Table S1. Summary of the machine learning algorithms.

Algorithm name	Package	Parameters
Decision tree	rpart package 4.1.19	Default
Support Vector Machine	e1071 package 1.7.13	kernel: 'radial', gamma: 10^{-6} ~ 10^{-2}, cost: 10^{2} ~ 10^{6}
Naïve Bayes	e1071 package 1.7.13	Default
K Nearest Neighbors	kknn package 1.3.1	Default
Random Forest	randomForest package 4.7.1.1	Default
Penalized Logistic Regression	glmnet package 4.1.8	L1 penalty
Neural network	tensorflow 2.0	neurons: 4, activation function: ReLU, hidden layers: 2, learning rate: 0.01, dropout rate: 0.5, batch size: 100, epochs: 150
Extreme Gradient Boosting	xgboost package 1.7.5.1	nround: 300

Supplementary Methods

The construction of polygenic risk scores

The PD-PRS was calculated using the SDPR method[1]. SDPR assumes the model $y = X\beta + \epsilon$, where X is the genotypes, β is the effect sizes, and $\epsilon_i \sim N(0, \sigma_\epsilon^2)$. SDPR employs adaptive modeling with a normal mixture distribution and adjusts the likelihood function as $\hat{\beta}|\beta \sim N(R\beta, \frac{R}{N})$, where R is the reference linkage disequilibrium matrix. SDPR does not impose a specific parametric distribution on the effect sizes. Instead, it assumes that the effect size of the ith SNP β_i, follows a normal distribution with a mean of 0 and a variance of σ_β^2, and places a Dirichlet process prior on σ_β^2: $\beta_i \sim N\left(0, \sigma_\beta^2\right), \sigma_\beta^2 \sim DP\left(H_\beta, \alpha\right)$, where H_β is the base distribution and α is the concentration parameter controlling the shrinkage of the distribution on σ^2 toward H. Additionally, SDPR utilizes the block structure of the reference linkage disequilibrium matrix to implement a parallel and fast mixing Markov Chain Monte Carlo algorithm. This is used to obtain posterior samples for estimating the effect sizes. SDPR can be downloaded from http://github.com/eldronzhou/SDPR.

References

1. Zhou, G.; Zhao, H. A fast and robust Bayesian nonparametric method for prediction of complex traits using summary statistics. *PLoS Genet* **2021**, *17*, e1009697, doi:10.1371/journal.pgen.1009697.
